# Control of tick infestations and pathogen prevalence in cattle and sheep farms vaccinated with the recombinant Subolesin-Major Surface Protein 1a chimeric antigen

**DOI:** 10.1186/1756-3305-7-10

**Published:** 2014-01-08

**Authors:** Alessandra Torina, Juan A Moreno-Cid, Valeria Blanda, Isabel G Fernández de Mera, José M Pérez de la Lastra, Salvatore Scimeca, Marcellocalogero Blanda, Maria Elena Scariano, Salvatore Briganò, Rosaria Disclafani, Antonio Piazza, Joaquín Vicente, Christian Gortázar, Santo Caracappa, Rossella Colomba Lelli, José de la Fuente

**Affiliations:** 1 Intituto Zooprofilattico Sperimentale della Sicilia, 90129, Palermo, Sicily, Italy; 2 Faculty of Veterinary Medicine, Universitá degli Studi di Messina, Messina, Sicily, Italy; 3 SaBio. Instituto de Investigación en Recursos Cinegéticos IREC-CSIC-UCLM-JCCM, Ronda de Toledo s/n, 13005, Ciudad Real, Spain; 4 Department of Veterinary Pathobiology, Center for Veterinary Health Sciences, Oklahoma State University, Stillwater, Oklahoma 74078, USA

**Keywords:** Subolesin, Tick, Vaccine, *Anaplasma*, *Babesia*, *Theileria*, Bovine, Ovine

## Abstract

**Background:**

Despite the use of chemical acaricides, tick infestations continue to affect animal health and production worldwide. Tick vaccines have been proposed as a cost-effective and environmentally friendly alternative for tick control. Vaccination with the candidate tick protective antigen, Subolesin (SUB), has been shown experimentally to be effective in controlling vector infestations and pathogen infection. Furthermore, *Escherichia coli* membranes containing the chimeric antigen composed of SUB fused to *Anaplasma marginale* Major Surface Protein 1a (MSP1a) (SUB-MSP1a) were produced using a simple low-cost process and proved to be effective for the control of cattle tick, *Rhipicephalus (Boophilus) microplus* and *R. annulatus* infestations in pen trials. In this research, field trials were conducted to characterize the effect of vaccination with SUB-MSP1a on tick infestations and the prevalence of tick-borne pathogens in a randomized controlled prospective study.

**Methods:**

Two cattle and two sheep farms with similar geographical locations and production characteristics were randomly assigned to control and vaccinated groups. Ticks were collected, counted, weighed and classified and the prevalence of tick-borne pathogens at the DNA and serological levels were followed for one year prior to and 9 months after vaccination.

**Results:**

Both cattle and sheep developed antibodies against SUB in response to vaccination. The main effect of the vaccine in cattle was the 8-fold reduction in the percent of infested animals while vaccination in sheep reduced tick infestations by 63%. Female tick weight was 32-55% lower in ticks collected from both vaccinated cattle and sheep when compared to controls. The seroprevalence of *Babesia bigemina* was lower by 30% in vaccinated cattle, suggesting a possible role for the vaccine in decreasing the prevalence of this tick-borne pathogen. The effect of the vaccine in reducing the frequency of one *A. marginale msp4* genotype probably reflected the reduction in the prevalence of a tick-transmitted strain as a result of the reduction in the percent of tick-infested cattle.

**Conclusions:**

These data provide evidence of the dual effect of a SUB-based vaccine for controlling tick infestations and pathogen infection/transmission and provide additional support for the use of the SUB-MSP1a vaccine for tick control in cattle and sheep.

## Background

Tick infestations affect animal health and production worldwide, both for the impact on animal weight gain and milk production and for the pathogens transmitted by these ectoparasites [1-4]. Acaricides are a major component of integrated tick control strategies, but their application has had limited efficacy in reducing tick infestations and is often accompanied by serious drawbacks, including the selection of acaricide-resistant ticks, environmental contamination and contamination of milk and meat products with drug residues [4]. All of these issues reinforce the need for alternative approaches to control tick infestations and pathogen transmission, including the use of vaccines with tick antigens [5-7].

In the early 1990s, commercial vaccines containing the recombinant *Rhipicephalus (Boophilus) microplus* BM86 gut antigen were developed and commercialized for the control of cattle tick infestations [8]. These vaccines proved to be a cost-effective alternative for cattle tick control through the reduction of the number of engorged female ticks, their weight and reproductive capacity and the prevalence of some tick-borne pathogens [1,8]. However, BM86-based vaccines have limited efficacy against other tick species and thus new vaccines are needed for the control of multiple tick species infestations, which occur in many areas used for animal husbandry [5,6,8].

Recently, Subolesin (SUB) was discovered as a new candidate tick protective antigen [9,10]. Vaccination trials with recombinant SUB and its ortholog in insects, Akirin, demonstrated effective control of arthropod vector infestations in various hard and soft tick species, mosquitoes, sand flies, poultry red mites and sea lice by reducing their numbers, weight, oviposition, fertility and/or molting and also reduced tick infection with tick-borne pathogens, *Anaplasma phagocytophilum*, *Anaplasma marginale*, *Babesia bigemina* and *Borrelia burgdorferi*[11]. Furthermore, the chimeric antigen, tick SUB fused with *A. marginale* Major Surface Protein 1a (MSP1a; SUB-MSP1a), was produced in *Escherichia coli* using a simple and low-cost process. Use of a vaccine with bacterial membranes containing the SUB-MSP1a chimera with surface-exposed SUB provided control of *R. microplus* and *R. annulatus* tick infestations [12,13], and this vaccine formulation was proposed as a low-cost and effective alternative means of tick control.

However, vaccination trials with SUB-MSP1a were conducted under controlled conditions and only in cattle experimentally infested with *R. microplus* and *R. annulatus*[12], which limit the assessment of the potential impact of this vaccine for the control of tick infestations and the prevalence of tick-borne pathogens under field conditions. To address these limitations, herein we conducted field trials on cattle and sheep farms in order to assess the efficacy of the SUB-MSP1a vaccine for the control of multiple tick species and tick-borne pathogens.

## Methods

### Experimental design and rationale

The field trial was designed to characterize the effect of vaccination with SUB-MSP1a on tick infestations and the prevalence of tick-borne pathogens at the DNA and serological levels in a randomized controlled prospective study. Two cattle and two sheep farms with similar geographical locations and production characteristics were randomly assigned as control or vaccinated herds. These farms were followed for one year prior to vaccination and 9 months after vaccination. Vaccine trials were approved by the Italian Ministry of Health (Direzione Generale della Sanita’ Aimale e dei Farmaci Veterinari, permit no. DGSAF 0002336-P-08/02/2011).

### Study site

**Figure 1:**
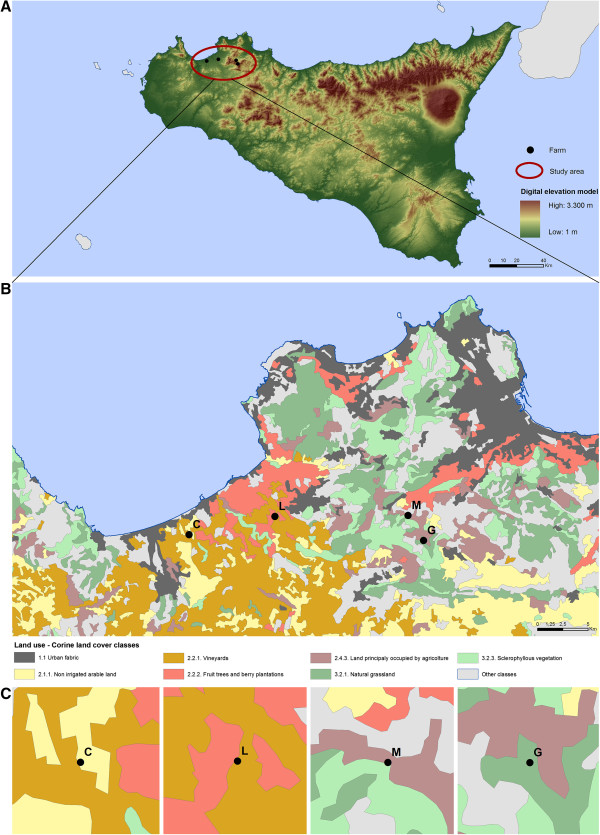
**Localization of cattle and sheep farms and land use in the study area.** Maps were constructed using the Esri ArcMap 9.3 software. **(A)** Localization of the study area in the Province of Palermo, Sicily. The digital elevation model was processed through the interpolation of level curves values of the Sicilian region, obtaining the elevations of study sites. **(B)** The land use of the areas near to the farms was obtained from Corine Land Cover 2006 processed by the European Environmental Agency describing the coverage and, in part, the use of the soil in Europe. Spatial selection allowed deriving the different levels of the land use classes that affect the areas where the farms are placed. **(C)** The analysis showed that vaccinated and control sheep (L and C) and cattle (M and G) farms are located close to each other in the same region and have similar land use.

Two cattle farms (identified as G for vaccinated and M for control) and two sheep farms (identified as C for vaccinated and L for control) located in the Province of Palermo, Sicily, were included in the trial (Figure 1). Cattle farms had a similar location (G, 38.00738 and 13.25156; M, 38.03039 and 13.23532), altitude (G, 950 m; M, 700 m), and number of animals (G, N = 35; M, N = 31). Sheep farms also had a similar location (C, 38.02188 and 12.98748; L, 38.03482 and 13.08531), altitude (C, 150 m; L, 175 m), and number of animals (C, N = 133; L, N = 123). Land use was also similar between cattle and sheep farms (Figure 1).

### Vaccine preparation and vaccination

Unless otherwise indicated, all reagents used in this work were purchased either from Sigma-Aldrich (St Louis, MO, USA) or VWR International Eurolab S.L. (Mollet del Vallés, Barcelona, Spain). The vaccine containing bacterial membranes with surface-exposed *R. microplus* SUB-MSP1a chimeric antigens was prepared as previously described [12]. Briefly, recombinant *E. coli* JM109 cells transformed with the pMBXAF3 expression vector were propagated in 1 litre flasks containing 250 ml Luria–Bertani (LB) broth supplemented with 10 g/l tryptone, 5 g/l yeast extract, 10 g/l NaCl, 50 μg/ml ampicillin and 0.4% glucose (Laboratorios CONDA S.A., Madrid, Spain) for 2 h at 37ºC and 200 rpm and then for 5.5 h after addition of 0.5 mM final concentration of isopropyl-β-d-thiogalactopyranoside (IPTG) for induction of recombinant protein production [14]. The cells were harvested by centrifugation at 10,000 x g for 15 min at 4ºC and then 1 g of cell pellet was resuspended in 5 ml of disruption buffer (100 mM Tris HCl, pH 7.5, 150 mM NaCl, 1 mM PMSF, 5 mM MgCl_2_ · 6H_2_O and 0.1% (v/v) Triton X-100) and disrupted using a cell sonicator (Model MS73; Bandelin Sonopuls, Berlin, Germany). After disruption, the insoluble protein fraction containing the membrane-bound SUB-MSP1a was collected by centrifugation at 21, 500 x g for 15 min at 4°C. The membrane-bound insoluble protein fraction containing over 50% of total proteins corresponding to the SUB-MSP1a chimera was resuspended in PBS, pH 7.4 and adjuvated in Montanide ISA 50 V2 (Seppic, Paris, France) at a concentration of 125 μg/ml.

All cattle and sheep present in the farms, including newborns at month 4 of age and adults imported during the trial were treated. Animals in cattle farm G and sheep farm C were vaccinated with two immunization doses of 1 ml containing 100 μg of the antigen preparation. Animals in cattle farm M and sheep farm L were injected with a similar volume of adjuvant/saline alone as control. Injections were done intramuscularly in the back of the animals using a 2.5-ml syringe and an 18G needle. Cattle in vaccinated and control farms were vaccinated or injected with adjuvant/saline on March 19^th^ and April 20^th^, 2012. Sheep in vaccinated and control farms were vaccinated or injected with adjuvant/saline on March 13^th^ and April 12^th^, 2012. Cattle in both vaccinated and control farms were treated with tilmicocin to prevent pneumonia prior to the first vaccination or adjuvant/saline injection following the manufacturer’s recommendations (TILMI-kel; KELA Laboratoria, Hoogstraten, Belgium). No contraindications have been described for this or similar products with respect to response to vaccination.

### Sample collection

In each farm, ticks and EDTA-treated and untreated blood samples were collected from all animals before each immunization and four weeks after the last immunization and then monthly from randomly selected individuals representing 10% of the animals present in the farm. Serum was separated from blood samples by centrifugation and stored with EDTA-treated blood samples at −20°C.

### Characterization of tick infestations

Collected ticks were counted for each animal, identified by morphological features using standard taxonomic keys for Italian Ixodidae [15] and preserved in 70% ethanol. Replete female ticks were weighed individually and the weights (mg) were compared between animals in the vaccinated farm before and after vaccination and between vaccinated and control farms by Student’s *t*-test with unequal variance (p = 0.05). Tick infestations (ticks/animal) were modeled separately for cattle and sheep using a generalized lineal model with binomial function and logit error with the dependent variables presence/absence of ticks and sampling time and farm as explanatory variables (p = 0.01; SPSS Statistics version 19, Surrey, UK). Tick infestations (ticks/animal) were compared between vaccinated and control animals using an ANOVA test (p = 0.05). The percent of animals infested with ticks before and after vaccination was compared between vaccinated and control farms by Student’s *t*-test with unequal variance (p = 0.05).

### Pathogen DNA identification by PCR

DNA was extracted from EDTA-treated blood samples using the PureLink Genomic Mini kit (Invitrogen, Carlsbad, CA, USA) following the manufacturer’s instructions. DNA samples were analyzed by PCR as reported previously to detect the presence of DNA from *Anaplasma* spp. [16] in all samples and positive samples were then analyzed for *A. marginale/A. ovis*[17,18] and *A. phagocytophilum*[19] DNA. The presence of DNA from *A. marginale*[17,18]*, Babesia bovis*[20], *B. bigemina*[20] and *Theileria annulata*[21] was analyzed in cattle only while DNA from *A. ovis*, *B. ovis*[22] and *T. ovis*[23] was characterized in sheep only. *Coxiella burnetii*[24] and *A. phagocytophilum*[19] DNA was characterized in both cattle and sheep samples.

PCRs were performed in a reaction buffer containing 1.5 mM MgCl_2_, 0.2 mM dNTPs, forward and reverse primers at a concentration of 0.4 mM, and 0.025 U/μl of Taq polymerase (5 U/μl) (Promega, Madison, WI, USA). For each reaction, a positive control consisting of pathogen DNA and a negative control in which DNA was replaced by water were used. PCR products were visualized after agarose gel electrophoresis containing 10 μg/ml ethidium bromide. Pathogen DNA prevalence (% positive animals) was compared between animals in the vaccinated farm before and after vaccination and between vaccinated and control farms by Student’s *t*-test with unequal variance (p = 0.05).

### Serological analyses

Serum antibody titers were determined using antigen-specific indirect ELISAs against SUB-MSP1a or SUB [12,25]. Briefly, purified antigens (0.1 μg/well) were used to coat ELISA plates overnight at 4°C. Plates were then washed three times with PBS/0.1% tween 20, pH 7.2. Sera were serially diluted to 1:100 and 1:1000 in PBS/0.5% Tween 20, pH 7.2 (PBST) and 10% fetal bovine serum (Sigma). The plates were incubated with the diluted sera for 1 hr at 37°C, washed three times with PBST and then incubated with 1:10,000 rabbit anti-bovine immunoglubolin G (IgG)-horseradish peroxidase conjugate (Sigma) for 1 hr at 37°C. Plates were washed three times with PBST and the color reaction was developed after incubation at 37°C with 200 μl of the substrate SIGMAFAS™OPD (Sigma). The reaction was stopped after 20 min with a solution of 4N sulphuric acid and the O.D._450nm_ was determined. Antibody titers were considered positive when they yielded an O.D._450nm_ value at least twice as high as the preimmune serum. Antibody titers were expressed as the O.D._450nm_ value for the highest serum dilution (1:1000) and compared between vaccinated and placebo control cattle using an ANOVA test (p = 0.05).

Bovine and ovine serum samples were analyzed using commercial ELISA kits for the presence of antibodies against *A. marginale*/*A. ovis* (VMRD, Pullman, WA, USA), *B. bigemina* (Svanova Biotech AB, Uppsala, Sweden) and *C. burnetii* (ID.vet, Montpellier, France) following manufacturer’s recommendations. The presence of antibodies against *T. annulata* was evaluated by immunofluorescence using antigen slides prepared as described previously [26]. Pathogen seroprevalence (% positive animals) was compared between animals in the vaccinated farm before and after vaccination and between vaccinated and control farms by Student’s *t*-test with unequal variance (p = 0.05).

### PCR amplification and sequencing of *A. marginale* and *A. ovis* major surface protein 4 (*msp4*) gene

Total DNA was extracted from EDTA-treated blood samples and analyzed by *msp4* PCR as described before [17] in all samples positive for *Anaplasma* spp. DNA. The *msp4* amplicons were purified using the MinElute PCR Purification Kit (Qiagen, Hilden, Germany) and sequenced (Secugen S.L., Madrid, Spain). Genotype frequencies were calculated as the percent of animals positive for each genotype. A correlation analysis was conducted to analyze genotype frequencies in time using Excel. The *msp4* sequences were aligned using the program AlignX (Vector NTI Suite V 5.5, InforMax, North Bethesda, MD, USA)*.* The *msp4* sequences were submitted to the GenBank under accession numbers [GenBank: KF739427-KF739433].

## Results and Discussion

### Antibody response in cattle and sheep vaccinated with the SUB-MSP1a antigen

**Figure 2:**
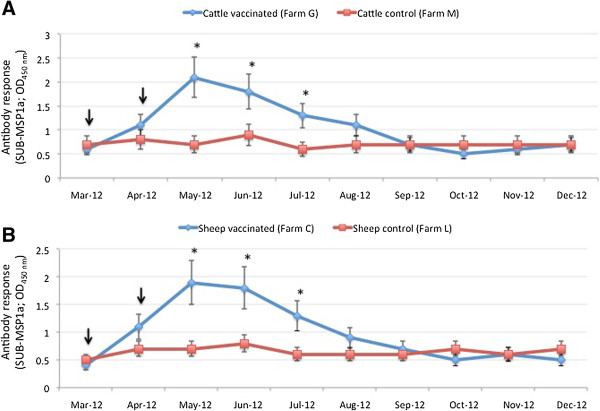
**Antibody response in cattle and sheep.** Serum antibody titers to the recombinant vaccine antigen, SUB-MSP1a, were determined by ELISA in **(A)** cattle and **(B)** sheep. Antibody titers were expressed as the OD_450nm_ value for the 1:1000 serum dilution, represented as Ave ± SD and compared between vaccinated and control animals using an ANOVA test (*p < 0.05). The time of immunization shots are indicated with arrows.

Cattle and sheep from vaccinated herds developed antibodies against SUB-MSP1a after vaccination, reaching a peak one month after the last immunization (Figure 2A and B). Significantly higher antibody titers against the vaccine antigen, SUB-MSP1a (Figure 2A and B) or recombinant SUB [Additional file 1: Figures S1 and S2] persisted in vaccinated animals for three months after the last immunization when compared to control animals. These results were similar to those previously obtained in cattle vaccinated with SUB-MSP1a [12]. Antibody titers to MSP1a were not determined because they are irrelevant for the studies reported here in part because antibodies in livestock persistently infected with *Anaplasma* react with MSP1a, which would preclude assessment of the humoral response to this part of the antigen after vaccination.

### Effect of vaccination on tick infestations on cattle and sheep

**Figure 3:**
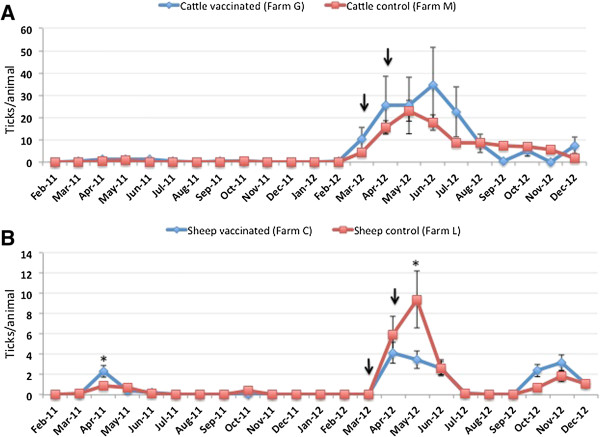
**Tick infestations in cattle and sheep.** Ticks found on animals in both vaccinated and control **(A)** cattle and **(B)** sheep farms were counted and stored in 70% ethanol. Tick infestations (ticks/animal) were represented as Ave ± SD and compared between vaccinated and control animals using an ANOVA test (*p < 0.05). The time of immunization shots are indicated with arrows.

One of the effects of SUB-MSP1a and other tick vaccines is the reduction in tick infestations [5,8,10,12]. The tick infestation rate (ticks/animal) was very low in the year before vaccination with no differences between cattle farms (Figure 3A; generalized lineal model, p = 0.3) but did differ between sheep farms with higher tick infestations in farm C (Figure 3B; generalized lineal model, p < 0.0001). However, tick infestations were higher in the second year of the experiment (Figure 3A and B), probably reflecting year-to-year variations in tick populations that occur under natural conditions in Sicily [27]. Differences were not observed in tick infestation rates between vaccinated and control cattle for both total tick counts (Figure 3A; generalized lineal model, p = 0.09) and female tick counts [Additional file 1: Figure S3]. However, total tick counts (Figure 3B; generalized lineal model, p = 0.003) and female tick counts [Additional file 1: Figure S4] per animal were lower by 63% and 60%, respectively in vaccinated sheep when compared to control animals one month after the last immunization. These results suggested differences between cattle and sheep that could be explained by different factors such as higher tick infestations in cattle when compared to sheep (Figure 3A and B) that require more time for the vaccine to reduce tick infestations, differences in tick species infesting cattle and sheep and/or other factors.

**Figure 4:**
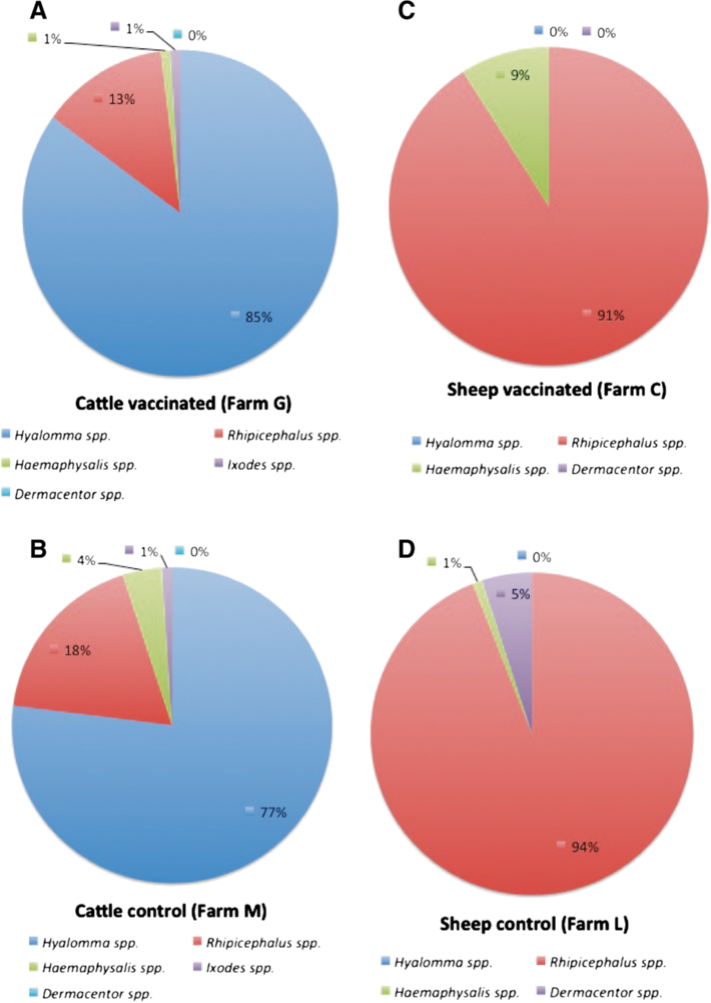
**Tick species infesting cattle and sheep.** Ticks collected from **(A, B)** cattle and **(C, D)** sheep in both **(A, C)** vaccinated and **(B, D)** control farms were classified, grouped according to tick genera and represented as percent of total ticks collected throughout the experiment.

Ticks collected on vaccinated and control animals throughout the experiment were classified and included *Hyalomma lusitanicum*, *Haemaphisalis punctata*, *Rhipicephalus bursa*, *Rhipicephalus sanguineus*, *Rhipicephalus turanicus*, *Rhipicephalus annulatus* and *Ixodes ricinus* in cattle, and *Dermacentor marginatus*, *R. sanguineus*, *R. turanicus* and *Haemaphisalis sulcata* in sheep. To address the differences between ticks infesting cattle and sheep, ticks were grouped according to their genera (Figure 4A-D). *Hyalomma* spp., followed by *Rhipicephalus* spp. and *Haemaphysalis* spp. were the predominant tick species found on cattle (Figure 4A and B). However, the predominant tick species infesting sheep were *Rhipicephalus* spp. followed by *Haemaphysalis* spp. and *Dermacentor* spp. (Figure 4C and D). These tick species are among the most abundant species found in the study area and the results reflect differences between preferred hosts for these species [27,28].

**Figure 5:**
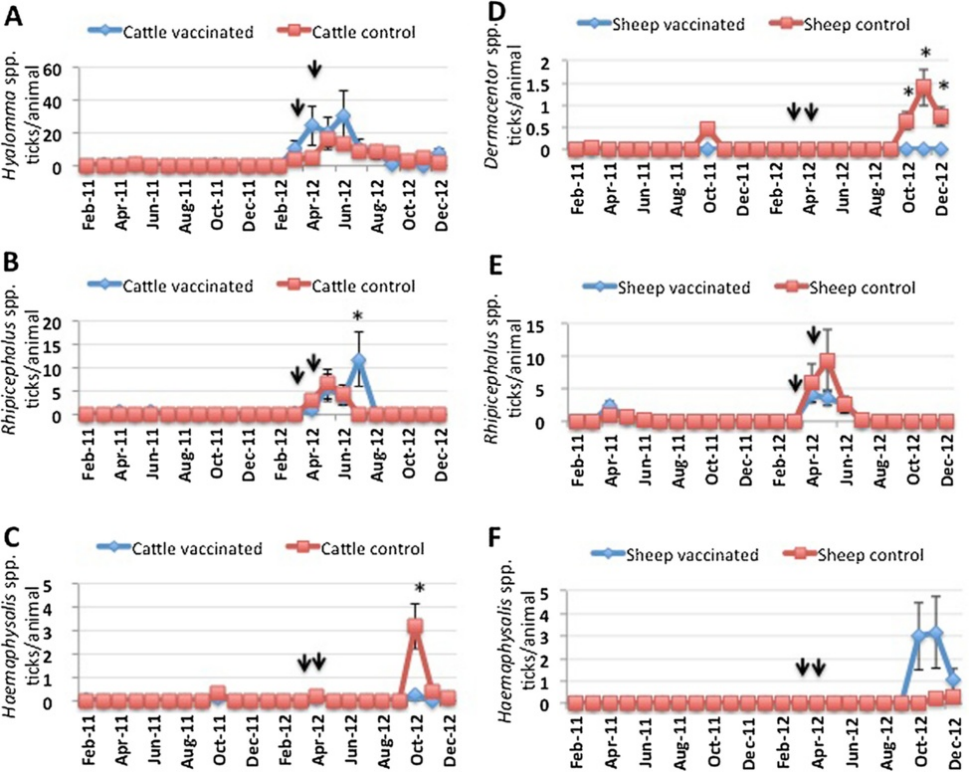
**Infestation with more abundant tick species in cattle and sheep.** The most abundant tick species found on **(A-C)** cattle and **(D-F)** sheep were counted, represented as ticks per animal (Ave ± SD) and compared between vaccinated and control animals using an ANOVA test (*p < 0.05). The time of immunization shots are indicated with arrows.

The tick infestation rate was then characterized for the most abundant tick species infesting cattle (Figure 5A-C) and sheep (Figure 5D-F). The appearance of the infestation peak varied between tick species reflecting variations in developmental seasonality for ticks [27]. In cattle, only *Haemaphysalis* spp. infestations were lower in vaccinated animals after vaccination (Figure 5C) while in sheep only *Dermacentor* spp. infestations were lower in vaccinated animals after vaccination (Figure 5D). Interestingly, *Rhipicephalus* spp. infestations were higher or similar in vaccinated cattle and sheep, respectively when compared to control animals (Figure 5B and E). At first, these results suggested a contradiction with previous experiments in which the SUB-MSP1a vaccine was protective against *R. microplus* and *R. annulatus* infestations in cattle [12]. However, the most abundant *Rhipicephalus* spp. collected from infested animals in this trial corresponded to *R. bursa*, *R. sanguineus* and *R. turanicus* in cattle and *R. sanguineus* and *R. turanicus* in sheep. These results suggested that the efficacy of the SUB-MSP1a vaccine differs between cattle and sheep and between different tick species (Figure 5A-F).

**Figure 6:**
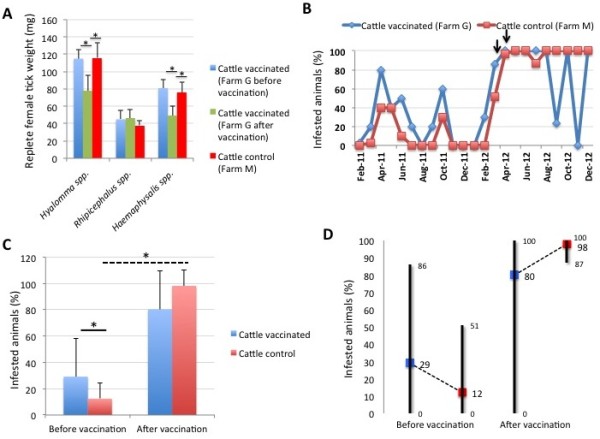
**Tick weight and percent infested cattle. (A)** Replete female ticks collected from vaccinated and control cattle were weighed, the weight (mg) represented as Ave ± SD and compared between cattle in the vaccinated farm before and after vaccination and between vaccinated and control cattle by Student’s *t*-test with unequal variance (*p < 0.05). **(B)** Percent infested cattle in vaccinated and control farms. The time of immunization shots are indicated with arrows. **(C)** Percent infested cattle before and after vaccination with SUB-MSP1a was represented as Ave ± SD and compared between vaccinated and control cattle by Student’s *t*-test with unequal variance (*p < 0.05). **(D)** Range values for the percent infested cattle in vaccinated and control farms before and after vaccination. Average values are shown to illustrate differences between vaccinated and control animals.

Previous results with the SUB-MSP1a vaccine showed that vaccination not only reduced cattle tick infestations but also the weight of replete female ticks [12]. Additionally, field application of BM86-based commercial vaccines showed a reduction in the percent of infested cattle [8]. Therefore, the effect of the vaccine was characterized on the weight of female ticks collected from cattle and sheep and the percent of infested animals in both control and vaccinated farms. The results showed *Hyalomma* spp. and *Haemaphysalis* spp. but not *Rhipicephalus* spp. female ticks collected from vaccinated cattle had significantly 32-39% lower weights when compared to the same animals before vaccination and to control cattle (Figure 6A). Furthermore, the percent of infested cattle was higher before vaccination but lower after vaccination in the vaccinated farm at some time points when compared to control animals (Figure 6B). The analysis of the number of infested cattle before and after vaccination showed that while the percent of infested animals was significantly higher in cattle in the vaccinated farm when compared to control cattle before vaccination, differences were not observed after vaccination between vaccinated and control cattle but only in the control farm before and after vaccination (Figure 6C). These results showed that vaccination with SUB-MSP1a maintained a similar percent of infested cattle after vaccination while in the control farm the percent of infested animals increased in more than 8-fold in the second year of the trial (Figure 6D).

**Figure 7:**
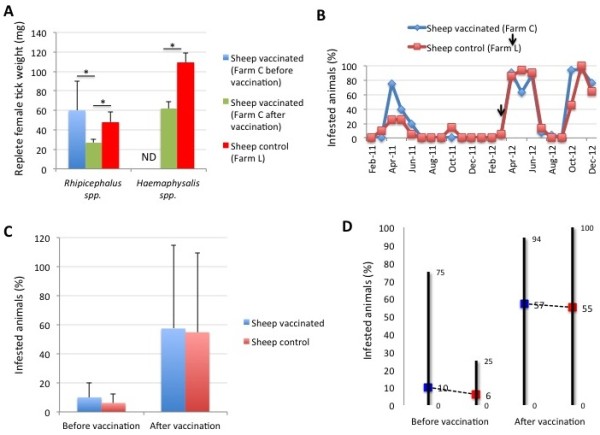
**Tick weight and percent infested sheep. (A)** Replete female ticks collected from vaccinated and control sheep were weighed, the weight (mg) represented as Ave ± SD and compared between sheep in the vaccinated farm before and after vaccination and between vaccinated and control sheep by Student’s *t*-test with unequal variance (*p < 0.05). **(B)** Percent infested sheep in vaccinated and control farms. The time of immunization shots are indicated with arrows. **(C)** Percent infested sheep before and after vaccination with SUB-MSP1a was represented as Ave ± SD and compared between vaccinated and control sheep by Student’s *t*-test with unequal variance (*p < 0.05). **(D)** Range values for the percent infested sheep in vaccinated and control farms before and after vaccination. Average values are shown to illustrate differences between vaccinated and control animals.

In sheep, 43-55% reduction in female tick weight was recorded for *Rhipicephalus* sp. and *Haemaphysalis* spp. collected from vaccinated animals when compared to the same animals before vaccination and to control sheep (Figure 7A). However, the percent of infested sheep was not affected by vaccination with SUB-MSP1a (Figure 7B-D).

Taken together, these results showed that the main effect of the SUB-MSP1a vaccine in cattle was the reduction in the percent of infested animals but not in the tick infestation rate of these animals. On the contrary, vaccination in sheep did not affect the percent of infested animals but reduced tick infestations. The reduction of female tick weight was observed in ticks collected from both vaccinated cattle and sheep.

### Effect of vaccination on the prevalence of tick-borne pathogens

The ultimate goal of tick vaccines is to reduce the prevalence of tick-borne diseases [7]. Tick vaccines have been shown to reduce the prevalence of tick-borne pathogens by reducing tick infestations and thus the exposure of susceptible hosts to pathogen transmission (e.g. BM86-based vaccine; [8]) and through reduction of tick vector capacity (e.g. SUB; [10,11]). Importantly, vaccination with SUB has shown that the vaccine reduces both tick infestations and pathogen infection/transmission, thus having a dual effect on reducing tick-borne diseases [11,29].

In our trial, the prevalence of tick-borne pathogens that has been described infecting cattle and/or sheep in Sicily [28,30-33] was analyzed at the DNA (*A. marginale*, *A. ovis*, *A. phagocytophilum*, *B. bovis*, *B. bigemina*, *B. ovis*, *T. annulata*, *T. ovis* and *C. burnetii*) and serology (*Anaplasma* spp., *B. bigemina*, *T. annulata* and *C. burnetii*) levels. The prevalence of *A. marginale, B. bovis*, *B. bigemina* and *T. annulata* was analyzed in cattle only while the prevalence of *A. ovis*, *B. ovis* and *T. ovis* was characterized in sheep only. *C. burnetii* and *A. phagocytophilum* prevalence were characterized in both cattle and sheep.

**Figure 8:**
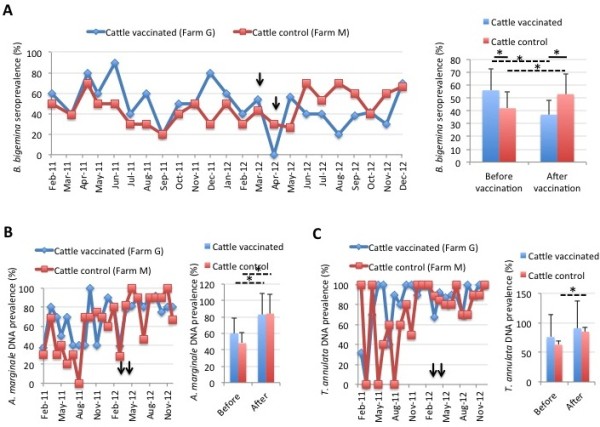
**Prevalence of tick-borne pathogens in cattle. (A)** The seroprevalence (%) of *B. bigemina* in vaccinated and control cattle was determined by ELISA, represented as Ave ± SD and compared between cattle in the vaccinated farm before and after vaccination and between vaccinated and control cattle by Student’s *t*-test with unequal variance (*p < 0.05). The time of immunization shots are indicated with arrows. **(B)** The DNA prevalence (%) for *A. marginale* in vaccinated and control cattle was determined by PCR, represented as Ave ± SD and compared between cattle in the vaccinated farm before and after vaccination and between vaccinated and control cattle by Student’s *t*-test with unequal variance (*p < 0.05). The time of immunization shots are indicated with arrows. **(C)** The DNA prevalence (%) for *T. annulata* in vaccinated and control cattle was determined by PCR, represented as Ave ± SD and compared between cattle in the vaccinated farm before and after vaccination and between vaccinated and control cattle by Student’s *t*-test with unequal variance (*p < 0.05). The time of immunization shots are indicated with arrows.

The results showed less than 1% DNA prevalence in both vaccinated and control cattle throughout the period of the trial for *A. phagocytophilum*, *B. bigemina*, *B. bovis* and *C. burnetii* (data not shown). However, *B. bigemina* seroprevalence decreased by 30% in vaccinated cattle after vaccination when compared to controls (Figure 8A), suggesting a role for the vaccine in reducing pathogen transmission. As expected for animals that develop persistent infection, the seroprevalence for *Anaplasma* spp. was higher than 90% throughout the experiment and did not change between vaccinated and control cattle nor before and after vaccination [Additional file 1: Figure S5]. For *A. marginale* DNA prevalence, the results showed an increase after vaccination but with similar levels for vaccinated and control cattle (Figure 8B). The seroprevalence for *C. burnetii* was less than 1% in cattle farm G throughout the experiment, while peaks of positive antibodies were observed in farm M but did not differ between vaccinated and control cattle nor before and after vaccination [Additional file 1: Figure S6]. The prevalence of *T. annulata* DNA increased after vaccination in control but not in vaccinated cattle but did not differ between vaccinated and control animals (Figure 8C). Similarly, *T. annulata* seroprevalence was high until the end of the experiment and did not differ between vaccinated and control cattle throughout the experiment [Additional file 1: Figure S7].

**Figure 9:**
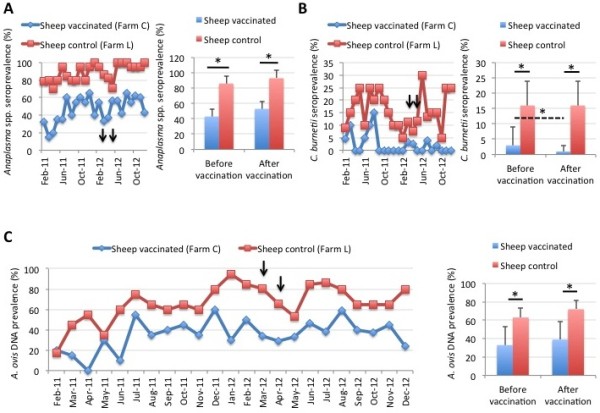
**Prevalence of tick-borne pathogens in sheep. (A)** The seroprevalence (%) of *Anaplasma* spp. in vaccinated and control sheep was determined by ELISA, represented as Ave ± SD and compared between sheep in the vaccinated farm before and after vaccination and between vaccinated and control sheep by Student’s *t*-test with unequal variance (*p < 0.05). The time of immunization shots are indicated with arrows. **(B)** The seroprevalence (%) of *C. burnetii* in vaccinated and control sheep was determined by ELISA, represented as Ave ± SD and compared between sheep in the vaccinated farm before and after vaccination and between vaccinated and control sheep by Student’s *t*-test with unequal variance (*p < 0.05). The time of immunization shots are indicated with arrows. **(C)** The DNA prevalence (%) for *A. ovis* in vaccinated and control sheep was determined by PCR, represented as Ave ± SD and compared between sheep in the vaccinated farm before and after vaccination and between vaccinated and control sheep by Student’s *t*-test with unequal variance (*p < 0.05). The time of immunization shots are indicated with arrows.

In sheep, less than 1% DNA prevalence was observed for *A. phagocytophilum*, *B. ovis* and *C. burnetii* in both vaccinated and control animals throughout the experiment (data not shown). The seroprevalence for *Anaplasma* spp. was higher in control than in vaccinated sheep but both before and after vaccination (Figure 9A). *C. burnetii* seroprevalence was also higher in control than in vaccinated sheep but decreased by 37% in vaccinated sheep after vaccination (Figure 9B). In agreement with serological results, *A. ovis* DNA prevalence was higher in control than in vaccinated sheep but both before and after vaccination (Figure 9C). The prevalence of *T. ovis* DNA was high throughout the experiment with no differences between vaccinated and control sheep [Additional file 1: Figure S8].

In summary, the results of vaccination with SUB-MSP1a on the prevalence of tick-borne pathogens suggested a role for the vaccine in decreasing *B. bigemina* seroprevalence in cattle. This finding is in agreement with the results of previous studies and demonstrated that vaccination with SUB reduces *B. bigemina* DNA levels in ticks fed on infected and vaccinated cattle [25,29]. Additionally, this result may reflect the reduction in the percent of infested animals shown in cattle vaccinated with SUB-MSP1a. However, in contrast to previous results in cattle vaccinated with SUB [25,29], vaccination with SUB-MSP1a did not reduce the prevalence for *A. marginale*. In sheep, the only effect of the vaccine was on the decrease of *C. burnetii* seroprevalence in vaccinated but not in control animals after vaccination. However, this result may reflect better farm management and animal health conditions in sheep farm C when compared to farm L, factors known to affect the prevalence of *C. burnetii*[34,35] and other tick-borne pathogens [36]. As shown in field trials using BM86-based commercial vaccines [1], the reduction in tick infestations in sheep vaccinated with SUB-MSP1a would require several years of vaccination before it could decrease the prevalence of some tick-borne pathogens.

### Characterization of the *A. marginale* and *A. ovis msp4* genotypes in cattle and sheep

*A. marginale* and *A. ovis* are transmitted not only biologically by ticks, but also mechanically by biting insects and blood contaminated fomites [37]. Therefore, despite the fact that vaccination with SUB-MSP1a did not affect *Anaplasma* spp. prevalence in this trial, we characterized *A. marginale* and *A. ovis msp4* genotypes trying to identify a possible effect of the vaccine on some genotypes likely transmitted by ticks. For this analysis, we used the *msp4* genetic marker, which has been used before for the characterization of genetic diversity in these species [17].

**Figure 10:**
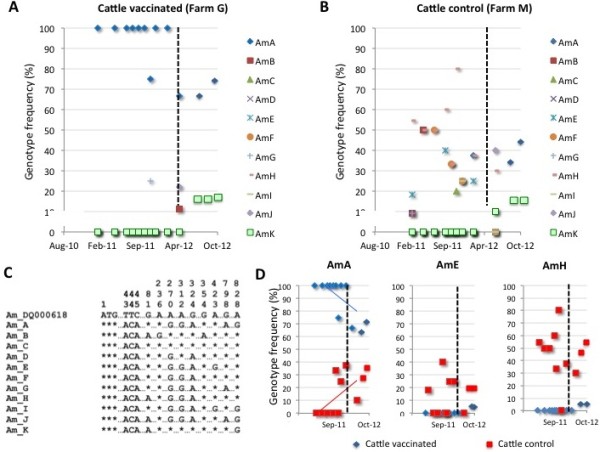
**Characterization of the *A. marginale msp4 *genotypes in cattle.** The *A. marginale msp4* coding region was amplified by PCR, sequenced and the frequency (%) for each genotype represented in **(A)** vaccinated and **(B)** control cattle. Dashed line represents time of last immunization shot. **(C)** The *msp4* sequences of each genotype were aligned and compared to the reference sequence (GenBank accession number DQ000618). Sequence positions (the adenine in the translation initiation codon ATG corresponds to position 1) with polymorphisms were identified. Asterisks represent sequence positions identical to the reference sequence. **(D)** Distribution in the frequencies of the most abundant *A. marginale msp4* genotypes in vaccinated and control cattle. Dashed line represents time of last immunization shot. A correlation analysis was conducted to analyze genotype frequencies in time using Excel and represented only when the correlation coefficient (R^2^) was ≥0.5.

**Figure 11:**
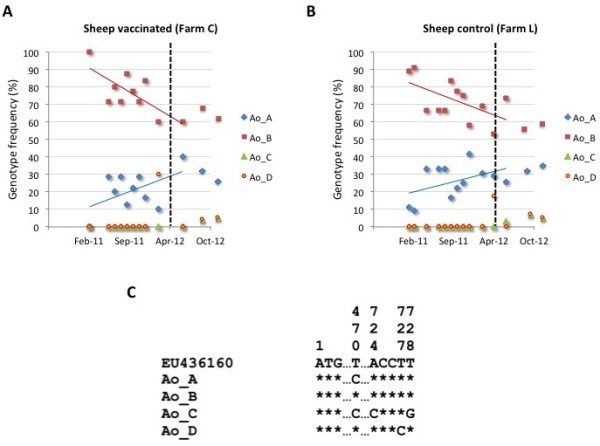
**Characterization of the *A. ovis msp4 *genotypes in sheep.** The *A. ovis msp4* coding region was amplified by PCR, sequenced and the frequency (%) for each genotype represented in **(A)** vaccinated and **(B)** control sheep. Dashed line represents time of last immunization shot. A correlation analysis was conducted to analyze genotype frequencies in time using Excel and represented only when the correlation coefficient (R^2^) was ≥0.5. **(C)** The *msp4* sequences of each genotype were aligned and compared to the reference sequence (GenBank accession number EU436160). Sequence positions (the adenine in the translation initiation codon ATG corresponds to position 1) with polymorphisms were identified. Asterisks represent sequence positions identical to the reference sequence.

The results showed a high genetic diversity for *A. marginale* in cattle (11 *msp4* genotypes; Figure 10A-C), a finding common to cattle herds in Sicily [38] and other regions of the world [39,40]. Genotype frequencies were different between vaccinated and control farms and between samples collected before and after vaccination (Figure 10A and B). Interestingly, the analysis of the most frequent genotypes showed an effect of cattle vaccination with SUB-MSP1a on decreasing the frequency for genotype AmA, but not for AmE and AmH genotypes (Figure 10D). These results suggested an effect of the vaccine in reducing the frequency for *A. marginale* genotype AmA, probably reflecting the reduction in the percent of tick-infested animals shown in cattle vaccinated with SUB-MSP1a that reduces the prevalence of a tick-transmitted strain. As in previous studies [39,41], the genetic diversity for *A. ovis* (4 *msp4* genotypes) was lower when compared to *A. marginale* (Figure 11A-C). Differences were not observed in *A. ovis msp4* genotype frequency between vaccinated and control sheep nor before and after vaccination with SUB-MSP1a (Figure 11A and B).

## Conclusions

To our knowledge, this is the first vaccine trial assessing the control of multiple tick species infestations and pathogen prevalence in cattle and sheep and the first field trial using the SUB-MSP1a vaccine. The main findings of these studies were that (a) both cattle and sheep developed antibodies in response to vaccination; (b) the main effect of the vaccine in cattle was the reduction in the percent of infested animals but not in the tick infestation rate in these animals; (c) vaccination in sheep did not affect the percent of infested animals but reduced tick infestations; (d) female tick weight was lower in ticks collected from both vaccinated cattle and sheep when compared to controls; (e) lower *B. bigemina* seroprevalence in vaccinated cattle suggested a role for the vaccine in decreasing the prevalence of this tick-borne pathogen; and (f) the effect of the vaccine in reducing the frequency of one *A. marginale* genotype probably reflected the reduction in the prevalence of a tick-transmitted strain as a result of the reduction in the percent of tick-infested cattle. The lower female tick weights are likely to impact and decrease tick populations over time because it has been well documented that lower female tick engorgement weights correlate with the oviposition of smaller egg masses. This trend would likely reduce tick populations over time. As has been noted previously, tick vaccines are likely to be an important component of integrated tick control methods and would likely reduce the use of acaricides.

These results provide new evidence to support that SUB-based vaccines have the dual effect of controlling tick infestations and pathogen infection/transmission, thus reducing tick populations and their vector capacity to impact on the control of tick-borne diseases [42]. These results also provide additional support for the use of the vaccine containing *E. coli* membranes with the surface-exposed SUB-MSP1a chimera as a low-cost and effective alternative for tick control in cattle and sheep, even under conditions with multiple tick species infestations and the prevalence of several tick-borne pathogens.

## Competing interests

The authors declare that they have no competing interests.

## Authors’ contributions

JF, AT, SC, CG, RCL conceived the study and participated in its design and coordination. JF, AT drafted the manuscript. JAM-C prepared recombinant antigen and vaccine formulations. VB, IGFM, MES carried out the molecular genetic studies, participated in the sequence alignment and in drafting the manuscript. JMPL, MES carried out the immunoassays. JV performed the statistical analysis. SS collected, classified and weighed ticks. SS, SB, RD, and AP performed sample collection and processing. MB analyzed the localization of cattle and sheep farms and land use in the study area. All authors read and approved the final manuscript.

## Supplementary Material

Additional file 1 Additional information on host antibody response, tick infestations and pathogen prevalence. Supplementary figures. Click here for file
