# Supplementary material for: Control of tick infestations and pathogen prevalence in cattle and sheep farms vaccinated with the recombinant Subolesin-Major Surface Protein 1a chimeric antigen
Source: Parasit Vectors. 2014 Jan 8;7:10. doi: 10.1186/1756-3305-7-10 (PMC3896746; doi:10.1186/1756-3305-7-10)
Supplement: Additional file 1 — Additional information on host antibody response, tick infestations and pathogen prevalence. Supplementary figures. [file 1756-3305-7-10-S1.pdf]

Supplementary figures.

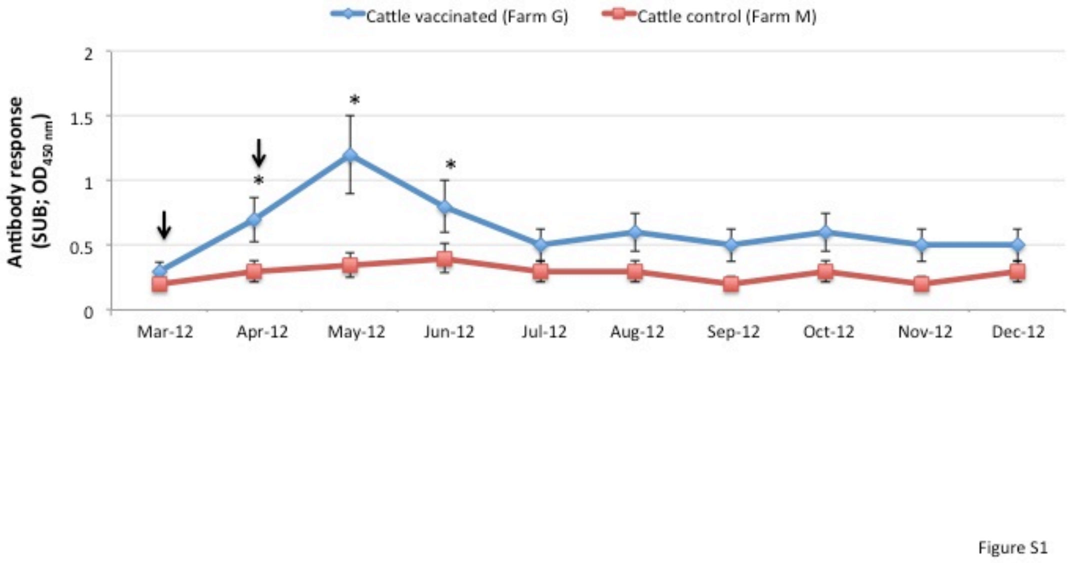

Figure S1. Antibody response in cattle. Serum antibody titers to recombinant SUB were determined by ELISA in cattle. Antibody titers were expressed as the OD_{450nm} value for the 1:1000 serum dilution, represented as Ave±SD and compared between vaccinated and control animals using an ANOVA test (*$p<0.05$). The time of immunization shots are indicated with arrows.

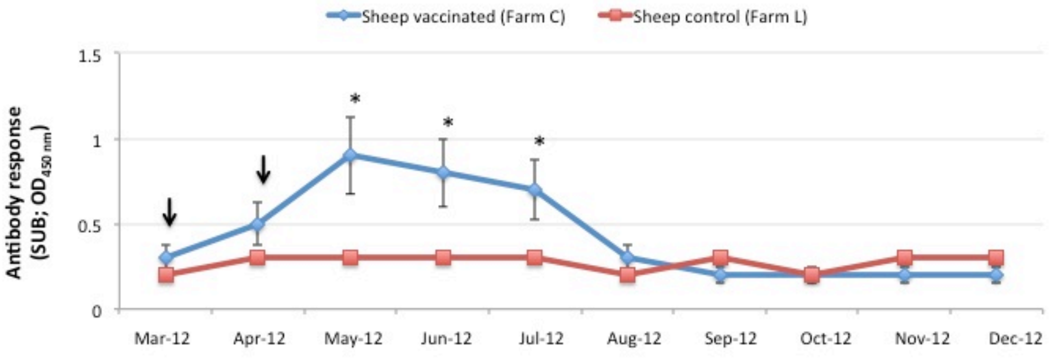

Figure S2

Figure S2. Antibody response in sheep. Serum antibody titers to recombinant SUB were determined by ELISA in sheep. Antibody titers were expressed as the OD_{450nm} value for the 1:1000 serum dilution, represented as Ave±SD and compared between vaccinated and control animals using an ANOVA test (*$p<0.05$). The time of immunization shots are indicated with arrows.

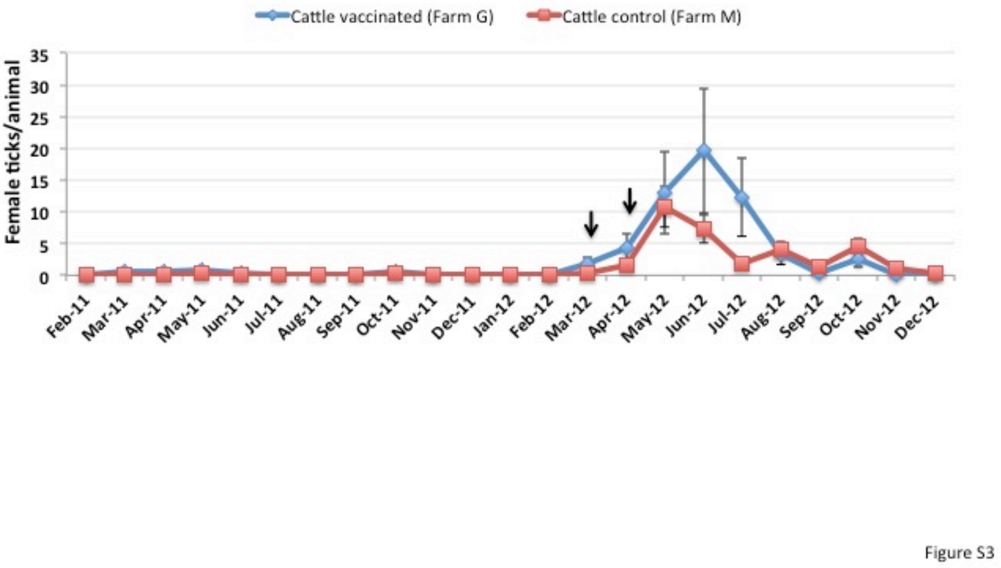

Figure S3. Tick infestations in cattle. Female ticks found on animals in both vaccinated and control cattle farms were counted and stored in 70% ethanol. Tick infestations (female ticks/animal) were represented as Ave±SD and compared between vaccinated and control animals using an ANOVA test ($p>0.05$). The time of immunization shots are indicated with arrows.

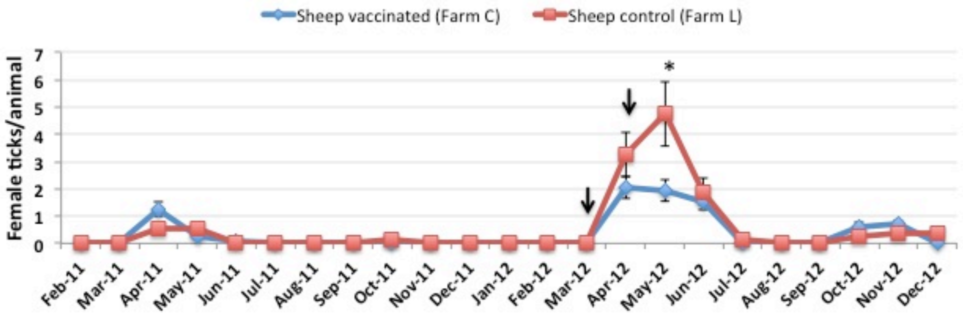

Figure S4

Figure S4. Tick infestations in sheep. Female ticks found on animals in both vaccinated and control sheep farms were counted and stored in 70% ethanol. Tick infestations (female ticks/animal) were represented as Ave±SD and compared between vaccinated and control animals using an ANOVA test (*$p<0.05$). The time of immunization shots are indicated with arrows.

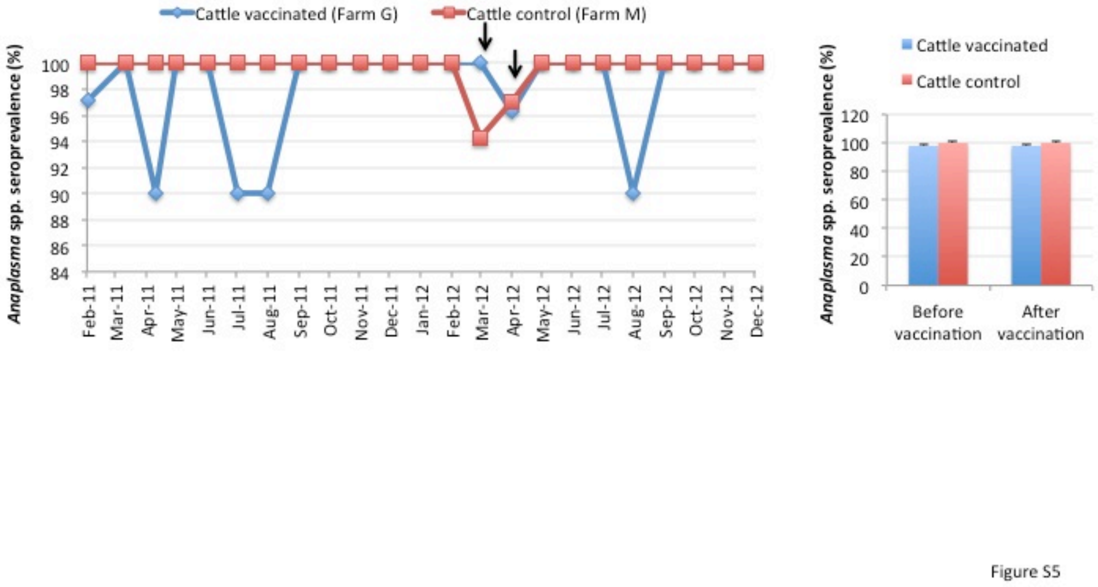

Figure S5. Seroprevalence of *Anaplasma* spp. in cattle. The seroprevalence (%) of *Anaplasma* spp. in vaccinated and control cattle was determined by ELISA, represented as Ave±SD and compared between cattle in the vaccinated farm before and after vaccination and between vaccinated and control cattle by Student's t-test with unequal variance ($p>0.05$). The time of immunization shots are indicated with arrows.

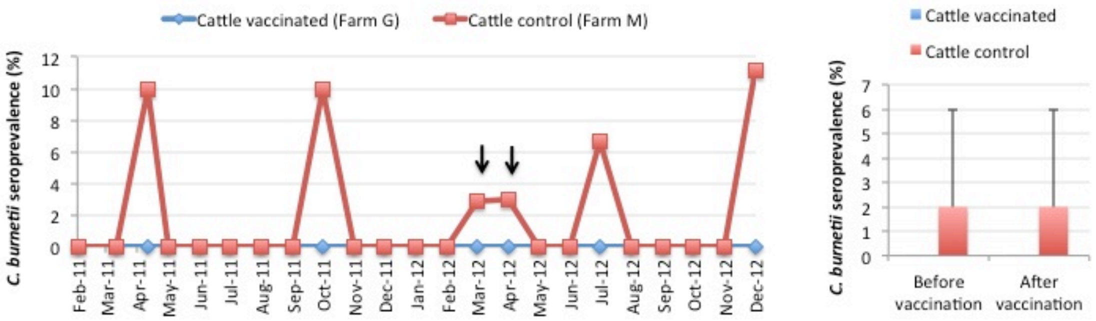

Figure S6

Figure S6. Seroprevalence of *C. burnetii* in cattle. The seroprevalence (%) of *C. burnetii* in vaccinated and control cattle was determined by ELISA, represented as Ave±SD and compared between cattle in the vaccinated farm before and after vaccination and between vaccinated and control cattle by Student's t-test with unequal variance ($p>0.05$). The time of immunization shots are indicated with arrows.

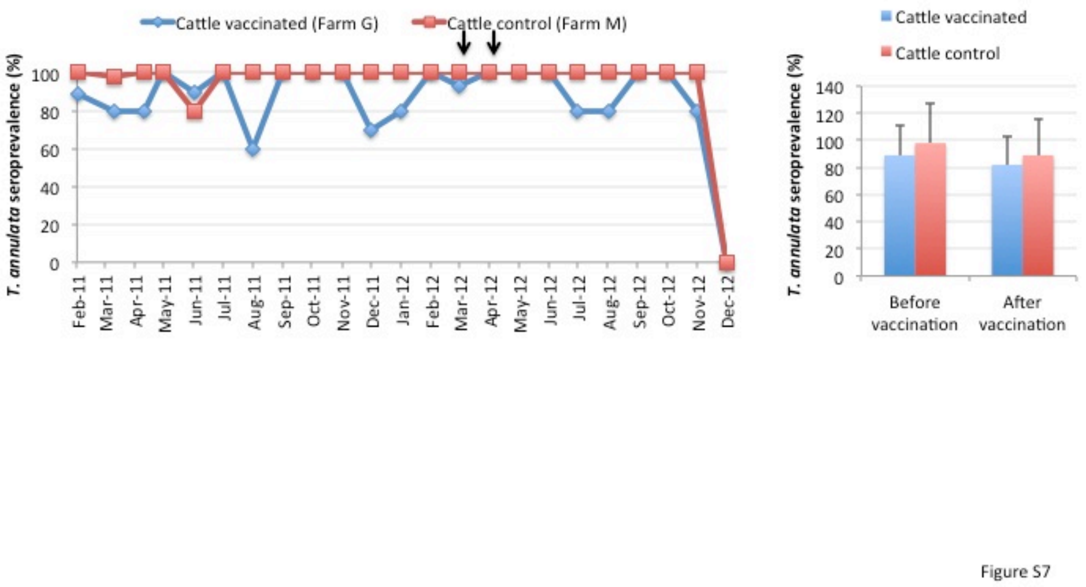

Figure S7. Seroprevalence of *T. annulata* in cattle. The seroprevalence (%) of *T. annulata* in vaccinated and control cattle was determined by immunofluorescence, represented as Ave±SD and compared between cattle in the vaccinated farm before and after vaccination and between vaccinated and control cattle by Student's t-test with unequal variance ($p>0.05$). The time of immunization shots are indicated with arrows.

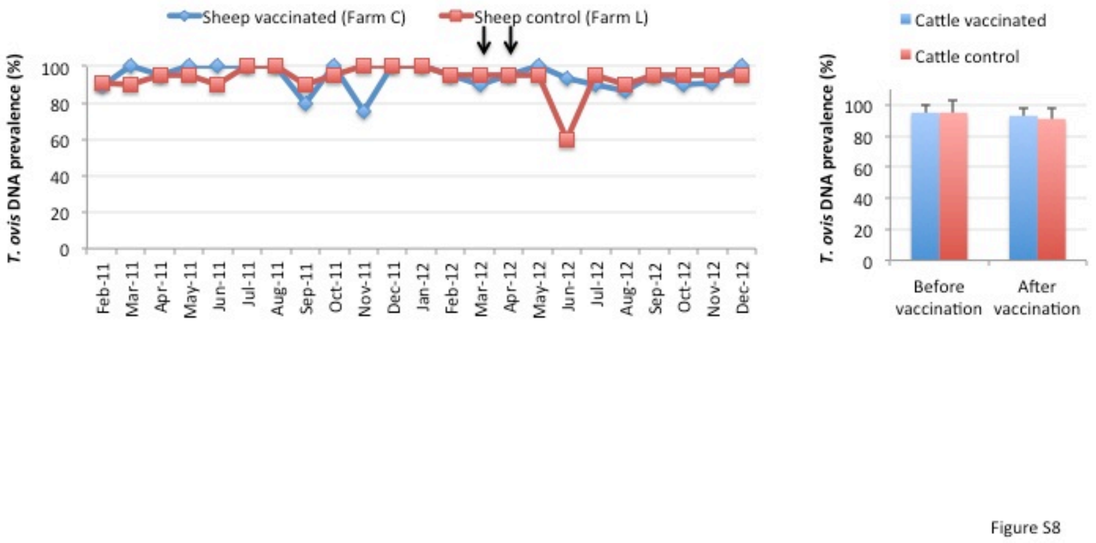

Figure S8. DNA prevalence of *T. ovis* in sheep. The DNA prevalence (%) for *T. ovis* in vaccinated and control sheep was determined by PCR, represented as Ave±SD and compared between sheep in the vaccinated farm before and after vaccination and between vaccinated and control sheep by Student's t-test with unequal variance ($p>0.05$). The time of immunization shots are indicated with arrows.
